# A cross-sectional study of psychological distress, burnout, and the associated risk factors in hospital pharmacists in Japan

**DOI:** 10.1186/s12889-016-3208-5

**Published:** 2016-07-08

**Authors:** Yuji Higuchi, Masatoshi Inagaki, Toshihiro Koyama, Yoshihisa Kitamura, Toshiaki Sendo, Maiko Fujimori, Yosuke Uchitomi, Norihito Yamada

**Affiliations:** Department of Neuropsychiatry, Okayama University Graduate School of Medicine, Dentistry and Pharmaceutical Sciences, Okayama, Japan; Department of Neuropsychiatry, Okayama University Hospital, 2-5-1 Shikata-cho, Kita-ku, Okayama, 700-8558 Japan; Department of Clinical Pharmacy, Okayama University Graduate School of Medicine, Dentistry and Pharmaceutical Sciences, Okayama, Japan; National Institute of Mental Health, Center for Suicide Prevention, National Center for Neurology and Psychiatry, Kodaira, Tokyo Japan; Innovation Center for Supportive, Palliative and Psychosocial Care, National Cancer Center, Tsukiji, Chuo-ku, Tokyo, Japan

**Keywords:** Burnout, Compassion fatigue, Hospital pharmacist, Japan, Pharmaceutical care

## Abstract

**Background:**

Opportunities for face-to-face communication with patients is increasing in modern hospital pharmacist practice. This may impose new burdens on hospital pharmacists. We performed a cross-sectional study to examine the prevalence of psychological distress, burnout, and compassion fatigue among hospital pharmacists. We also investigated possible relevant factors, such as sex, years of experience, hospital size, interpersonal work hours, and personality traits related to communication.

**Methods:**

We mailed self-administered questionnaires to all pharmacists (*n* = 823) belonging to the prefectural society of hospital pharmacists in Japan. The questionnaires were the General Health Questionnaire (GHQ-12), Burnout (BO) and Compassion Fatigue and Secondary Traumatic Stress (CF/STS) subscales of the Professional Quality of Life Scale, the Autism Spectrum Quotient (AQ), and the Adult ADHD (attention deficit hyperactivity disorder) Self-Report Scale (ASRS). We examined associations between personality traits (AQ, ASRS) and psychological burden (GHQ-12, BO, CF/STS) using rank ANCOVA or multivariate logistic regression analyses.

**Results:**

Complete responses were obtained from 380 pharmacists (46.2 % response rate). A substantial number of participants obtained scores that were higher than the cutoff points of the GHQ-12 (54.7 %), BO (49.2 %), and CF/STS (29.2 %). The GHQ-12 scores were negatively affected by years of experience (*p* < 0.001), and positively affected by AQ (*p* < 0.001) and ASRS (*p* < 0.001) scores. The BO scores was positively affected by AQ (*p* < 0.001) and ASRS (*p* = 0.001) scores, while the CF/STS (*p* = 0.023) score was negatively affected by years of experience, and positively affected by AQ (*p* < 0.001) and ASRS (*p* < 0.001) scores.

**Conclusions:**

There is a high prevalence of psychological distress and work-related burnout/CF among hospital pharmacists. Additionally, two common personality traits, such as autistic-like traits and ADHD-like symptoms, which might be related to communication style, could increase the risk of psychological distress and burnout/CF. Early risk assessment and preventive interventions that are specialized for these characteristics could protect individuals with these specific traits from burnout.

## Background

In accordance with a paradigm shift towards a patient-focused rather than a disease-focused approach in clinical medicine, pharmacists are trained in both traditional drug-oriented services, such as pharmacy compounding, and patient-oriented services [1]. In modern pharmaceutical care, which is focused on improving safety, therapeutic outcomes, and patient quality of life [1], pharmacists may face new psychological challenges. Pharmacists may be exposed to the emotional responses of angry patients with aggression [2], be required to provide direct patient care under the direction of a physician [3], and facilitate the distribution of information and advice between patients and other healthcare practitioners [4]. Healthcare professionals, such as radiologists who have little contact with patients, have occupational stress, such as effort/reward imbalance, high job demands and limited autonomy, and low social support [5]. Therefore, pharmacists may suffer from the same type of occupational stress. Health professionals dealing directly with patients who are facing challenging medical conditions are often subject to strong emotional interactions in their work setting, and thus are likely to experience chronic emotional stress that can induce burnout [6]. As with other healthcare professionals, high requirements regarding interpersonal communication can impose physical and psychological burdens on pharmacists.

Based on recent developments in the field, we believe that burnout and compassion fatigue (CF) in pharmacists need to be reconsidered. These conditions can represent overwhelming psychological burdens, and can cause physical, mental, and emotional health difficulties [7]. Burnout is a prolonged response to chronic emotional and interpersonal stressors on the job [8]. Compassion fatigue describes the caregiver cost of caring for patients with chronic illness (i.e., patients who will never fully recover) [9]. A previous study reported that CF in pharmacists may be associated with medical errors, such as errors in management of medicines [10]. For medical staff in a cancer care center, as many as 44 % of inpatient workers were at high risk of burnout, while 37 % were at high risk of CF [7]. Psychological distress among medical staff is correlated with high levels of burnout and CF [11].

With regard to pharmacy staff, the need for better burnout prevention strategies and clarification of burnout-prone behaviors were established in the 1980s [12]. However, investigations of the prevalence of burnout, related factors, and associated neuropsychological traits among pharmacists have been limited. Several factors associated with burnout of pharmacists have been identified, including age, marital status, work experience, work contentment, and workload [13]. However, knowledge of the associations between burnout and specific behavioral or neuropsychological traits related to communication, which may induce burnout/CF, is lacking.

Therefore, in this study, we investigated two psychological traits that may be associated with burnout and CF, in addition to demographic variables. The first trait was the presence of autistic-like traits (ALTs), which can resemble a milder form of autism spectrum disorder (ASD). ALTs can signal individuals who might have difficulty with sociality and communication [14], but are within the “normality” spectrum [15]. The second trait was attention deficit hyperactivity disorder (ADHD). Individuals with ADHD often encounter problems with interpersonal relationships in their youth. The tendencies characteristic of ADHD are thought to remain into adulthood, and the adult prevalence of ADHD ranges between 3.4 % and 4.4 % [16]. Those with ASD or ADHD in their youth may later have impaired social adjustment [17, 18], but there are limited studies on this issue.

However, as a result of recommendations by the Ministry of Health, Labour and Welfare in Japan, the demand for a change to a patient-oriented service from drug-oriented work in the role of the pharmacist has become stronger. Additionally, a medical fee is charged when hospital pharmacists perform face-to-face explanations of the need for medication or injection of anti-neoplastic agents to patients with cancer under physicians’ instructions. A fee is also charged when pharmacists perform 20 h or longer a week of drug-related work in the hospital, including answering consultations from other health workers or explaining risks to patients. Therefore, we were concerned that this drastic change imposes a great deal of stress on pharmacists, particularly on those with these two psychological traits. We hypothesized that hospital pharmacists with ALT-and/or ADHD-like tendencies are less socially adaptive with respect to their work environment, and thus are especially susceptible to burnout/CF.

To the best of our knowledge, few reports have evaluated psychological distress, burnout, and the associated risk factors in hospital pharmacists. Accordingly, this study aimed to investigate the following: 1) the prevalence of psychological distress and burnout/CF among hospital pharmacists; 2) the extent of correlation of specific demographic characteristics (e.g., sex and years of experience) with psychological distress and burnout/CF; and 3) whether high levels of ALT-and ADHD-like symptoms contribute to psychological distress and burnout/CF in hospital pharmacists. More information on these issues could contribute to enhanced risk assessment. Additionally, new data may facilitate environmental adjustments and early educational prevention interventions in hospital pharmacies. High stress in pharmacists may endanger not only the physical and mental health of practitioners, but also patients’ safety [19].

## Methods

### Subjects

The participants in this study were certified pharmacists working at hospitals in patient care. All participants were members of the Okayama Prefectural Society of Hospital Pharmacists. After receiving approval from the appropriate pharmacy representatives, we mailed self-administered questionnaires to all of the pharmacists who met the inclusion criteria and informed the potential participants of the aims, methods, risks, and benefits of the study. The participants were asked to complete all questionnaires anonymously. With the approval of the Ethics Committee at Okayama University Hospital, we assumed that the return of the questionnaires constituted informed consent. In 2014, the Okayama Prefectural Society of Hospital Pharmacists officially reported that there were 823 hospital pharmacists with a median of 11 years of work experience after being qualified as a pharmacist, although data were missing for 10 individuals. The participants initially completed questions about their demographic and professional background, providing information on age, sex, number of beds in the hospital in which they worked, number of years since being qualified as a pharmacist, and number of mean hours of interpersonal work per week.

### Measurements

#### The general health questionnaire

The General Health Questionnaire (GHQ) was developed by Goldberg and Hillier in 1979 as a screening instrument for identifying psychological distress among adults in primary care settings, and evaluating anxiety and depression symptoms in individuals without psychiatric disorders [20]. In a World Health Organization study of the general population, the 12-item General Health Questionnaire (GHQ-12) produced results that were comparable with the original longer version of the GHQ [21]. The GHQ-12 has been well-validated for general Japanese adult populations [22]. The questionnaire measures 12 symptoms of psychiatric morbidity (e.g., concentration, sleep disturbances). Each item is scored as either 0 (less or no more than usual) or 1 (more or much more than usual), for a maximum total score of 12. Individuals with scores above the threshold of 4 are considered to suffer from psychiatric morbidity. We previously reported that many clinical oncologists in Japan with high levels of burnout had high scores of the GHQ-12 [23].

#### Professional quality of life scale

The Professional Quality of Life Scale V (Pro.QOL) was developed by Stamm [24]. This scale comprises 30 self-report measures with 10 items per subscale. Using a six-point Likert-type scale (0 = never to 5 = very often), respondents were asked to indicate how frequently each item was experienced in the previous 30 days. The scores for each subscale were obtained by summing the items. The subscales are as follows: (a) burnout (BO), feelings of hopelessness and difficulties dealing with work; (b) compassion fatigue/secondary traumatic stress (CF/STS), which measures work-related secondary exposure to extremely stressful events; and (c) compassion satisfaction (CS), which is the pleasure derived by an individual when they are satisfied with their job performance. We used the BO and CF/STS subscales, which reflect the negative aspects of interpersonal practice of pharmacists. Because the Japanese version has not been updated with respect to the newest version (5), we used an older version (4) according to the manual [25]. Unfortunately, the reliability or validity of this Japanese version has not been determined. Using the manual for version 4 as a reference, we set the following cutoff points: a high level for BO was 27 or greater and a high level for CF/STS was 17 or greater. We used these cutoff points for descriptive statistics only, and used continuous numbers for other statistical analyses, as per the recommendations of the original author [24].

#### Autistic-like traits

Subclinical autistic features can be present in healthy humans. To quantify the presence of autistic features in adults with normal intelligence, Baron-Cohen developed a self-report questionnaire called the Autism Spectrum Quotient (AQ) [26]. The AQ is composed of 50 items that are rated on a four-point Likert-type scale ranging from 1 (not at all) to 4 (very well). The AQ has a dichotomous scoring method (0-0-1-1) ranging from 0 to 50. We used a validated Japanese version of the AQ (AQ-J) [27] with a cutoff point of 33 or greater to evaluate ALTs in our participants. Although the AQ has not been extensively used to investigate healthcare providers, AQ scores have been associated with poor mental health among factory workers [28].

#### Adult symptoms of attention deficit hyperactivity disorder

The Adult ADHD Self-Report Scale (ASRS) is used to screen adults with ADHD. The ASRS was developed and translated into various languages, including Japanese, by the World Health Organization. This test evaluates the frequency of all 18 Criterion A symptoms of ADHD according to the Diagnostic and Statistical Manual of Mental Disorders, Text Revision, 4th edition [29]. The ASRS-v1.1 Screener is a short screening test that consists of six of the 18 questions included in the ASRS. The screener sums unweighted dichotomous responses and outperforms the full ASRS in terms of sensitivity (68.7 %), specificity (99.5 %), and total classification accuracy (97.9 %), with a cutoff point of 4 or higher [29]. Each item explores how often a particular symptom of ADHD has occurred over the past 6 months, as rated on a five-point scale. The response options, which range from 0 (never) to 4 (very often), are dichotomized. We chose to use this short version to evaluate major ADHD symptoms for its convenience and reliability.

#### Statistical analysis

All analyses were performed using IBM SPSS, version 22 (IBM Japan, Tokyo, Japan). In descriptive statistics, the prevalence of psychological distress and burnout/CF are shown with 95 % confidence intervals (CIs). A value of *p* < 0.05 was considered statistically significant. We used the Kolmogorov–Smirnov test to evaluate the distribution of variables. When analyzing the associations between burnout/CF and demographic factors, we used the chi-square test for categorical variables (e.g., sex) and Spearman’s correlation coefficient for continuous variables. Inventory scores were compared using Spearman’s correlations because these scores were not normally distributed.

We examined the association between high levels of ALTs or ADHD symptoms and psychiatric morbidity using multivariate logistic regression analysis without a distribution assumption. We entered parametric AQ and ASRS scores as independent variables and GHQ-12 scores as the dependent variable. Covariates included years of experience, number of hospital beds, interpersonal work hours, and categorically coded sex.

None of the variables were normally distributed. We chose to use robust rank-based significance tests because parametric methods can result in inaccurate values when assumptions of normality and homogeneity of variance are not possible [30]. We used a rank ANCOVA with ranked AQ, ranked ASRS, and categorically coded sex (male = 0, female = 1) as fixed factors, covarying for ranked demographic data (years of experience, number of hospital beds, and interpersonal work hours) as independent variables, and psychological measurements (Pro.QOL-BO, CF/STS) as dependent variables [31].

## Results

### Background characteristics

**Fig. 1:**
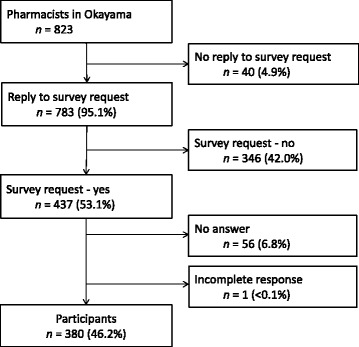
Study flowchart

All of the participants (*n* = 823) were certified pharmacists in Japan and were currently working in hospitals engaged in patient care. Ninety-two (59.7 %) among the 154 hospitals responded positively to our survey request and questionnaires were subsequently posted to 437 pharmacists. Complete responses were obtained from 380 pharmacists, yielding a response rate of 46.2 % (Fig. 1). A demographic summary of the respondents is shown in Table 1. There were 229 women (60.4 %) and the mean (± standard deviation) age of participants was 37.3 ± 10.8 years (missing data, *n* = 13). The median number of years since qualification as a pharmacist was 11. Forty-five (11.8 %) participants answered 0 to interpersonal working time. Based on background information, such as years of experience, we inferred that these participants were likely novice, management, or laboratory pharmacists.

**Table 1:** Demographic summary and descriptive statistics for questionnaire responses (*n* = 380)

Demographic Data	no.	%					
Women	229	60.4					
	Mean	Median	SD	Range			
Age (years, *n* = 13 data missing)	37.3	34	10.8	[24–66]			
Years after qualification	13.6	11	11.3	[0–44]			
Years after qualification, officially reported data (*n* = 823)	14.1	11	12.3	[0–50]			
Hospital beds	466.8	342	333.3	[19–1182]			
interpersonal work (hours per week)	11.3	8	11.3	[0–56]			
Questionnaire results	Mean	Median	SD	Observed range	Theoretical range	cut off	over cut off(%) [95 % CI]
GHQ-12	3.9	4	2.8	[0–12]	[0–12]	>3	54.7 [49.7 to 59.8]
Pro.QOL-BO	26	26	5.6	[8–39]	[0–50]	>26	49.2 [44.2 to 54.3]
Pro.QOL-CF/STS	13.5	13	6.1	[0–35]	[0–50]	>16	29.2 [24.6 to 33.8]
AQ	19.7	19	7.3	[5–41]	[0–50]	>32	5.5 [3.2 to 7.8]
ASRS	1.8	2	1.4	[0–6]	[0–6]	>3	11.6 [8.4 to 14.8]

*GHQ-12* the general health questionnaire-12, *AQ* the autism-spectrum quotient, *ASRS* the adult ADHD self-report scale v-1.1, *Pro.QOL* the professional quality of life scale, *BO* burnout *CF/STS* compassion fatigue/secondary traumatic stress, *CI* confidence interval

A substantial number of participants scored above the cutoff points of the GHQ-12 (54.7 %, 95 % CI: 49.7 to 59.8), Pro.QOL-BO (49.2 %, 95 % CI: 44.2 to 54.3), and Pro.QOL-CF/STS (29.2 %, 95 % CI: 24.6 to 33.8). A smaller number of participants scored above the clinical cutoff points of the AQ (5.5 %) and ASRS (11.6 %). Other data and descriptive statistics regarding the questionnaire responses are shown in Table 1.

### Correlation analysis of psychological measurements with demographic variables

Using the chi-square values and correlation coefficients (Spearman), we evaluated the associations between demographic characteristics and psychological measurements (GHQ-12 or Pro.QOL; Table 2). Because some data on age were missing, we omitted a correlation test involving age, and substituted years since qualification for this value. Sex, number of hospital beds, and number of interpersonal work hours were not significantly correlated with psychological measurements. However, the number of years since qualification was weakly, but significantly, negatively correlated with several psychological measures, including AQ, ADHD, CF/STS, and GHQ-12, but not BO, scores. Post-hoc subgroup analysis showed that years of experience also had a negative, but not significant, correlation with GHQ-12 scores in the group of high ALTs with the first quartile of AQ scores (data not shown).

**Table 2:** Correlation analysis of psychological measurement with demographic variables

	GHQ-12	Pro.QOL-BO	Pro.QOL-CF/STS
Gender (Chi Square^a^, *p* value)	*χ* ^2^ = 8.144, *p* = 0.774	*χ* ^2^ = 14.381, *p* = 0.993	*χ* ^2^ = 28.824,*p* = 0.719
Experienced Years (spearman’s r, *p* value)	***r*** **= −0.223,** ***p*** **< 0.001**	*r* = −0.034, *p* = 0.514	***r*** **= −0.116,** ***p*** **= 0.023**
Hospital Bed Number (spearman’s r, *p* value)	*r* = 0.017, *p* = 0.745	*r* = 0.027, *p* = 0.595	*r* = −0.004, *p* = 0.932
interpersonal work hours (spearman’s r, *p* value)	*r* = 0.007, *p* = 0.892	*r* = −0.032, *p* = 0.536	*r* = 0.054, *p* = 0.298
AQ (spearman’s r, *p* value)	***r*** **= 0.422,** ***p*** **< 0.001**	***r*** **= 0.248,** ***p*** **< 0.001**	***r*** **= 0.282,** ***p*** **< 0.001**
ASRS (spearman’s r, *p* value)	***r*** **= 0.345,** ***p*** **< 0.001**	***r*** **= 0.170,** ***p*** **= 0.001**	***r*** **= 0.284,** ***p*** **< 0.001**

bold: *p* < 0.05

^a^ The deviation of gender distribution in each score was analyzed in goodness-of-fit test

*AQ* the autism-spectrum quotient, *ASRS* the adult ADHD self-report scale v-1.1, *GHQ-12* the general health questionnaire-12, *Pro.QOL* the professional quality of life scale, *BO* burnout, *CF/STS* compassion fatigue/secondary traumatic stress

### Multivariate associations between AQ/ASRS and GHQ-12 scores, controlling for demographic variables

AQ (*p* < 0.001) and ASRS (*p* < 0.001) scores were significantly associated with psychiatric morbidity (Table 3). The Hosmer–Lemeshow test confirmed goodness of fit with respect to the logistic regression model (*p* > 0.05).

**Table 3:** Multivariate analyses using GHQ-12 cutoff sores as outcome variables: multivariate logistic regression analysis

	Psychiatric morbidity		
	OR	[95%CI]	*p* value
AQ: The Autism-Spectrum Quotient	**1.091**	**1.054–1.130**	***p*** **< 0.001**
ASRS: The Adult ADHD Self-Report Scale v-1.1	**1.392**	**1.170–1.657**	***p*** **< 0.001**
Experienced Years	0.982	0.961–1.003	*p* = 0.095
Hospital Bed Number	0.999	0.999–1.000	*p* = 0.098
Interpersonal Work Hours per week	1.009	0.989–1.029	*p* = 0.401
Gender (Women compared to men)	1.118	0.708–1.764	*p* = 0.633

Hosmer-Lemeshow test: 6.754, *p* = 0.563

bold: *p* < 0.05

GHQ cutoff score: above 3

*OR* odds ratio for one unit increase, *CI* confidence Interval

### Multivariate associations between AQ/ASRS and Pro.QOL scores with sex as a fixed factor, controlling for demographic variables

Before executing the rank ANCOVA, the homogeneity of covariance matrices were confirmed via Box’s Test of Equality of Covariance Matrices (*p* = 0.409) and Levene’s Test of Equality of Error Variances (ranked Pro.QOL-BO, *p* = 0.733; ranked Pro.QOL-CF/STS, *p* = 0.086). We were able to establish that the fixed factor and covariates were independent because we found no significant interaction effects between them. The results of the rank ANCOVA are shown in Table 4. Ranked AQ scores significantly affected ranked BO and ranked CF/STS scores, while ranked ASRS scores significantly affected ranked CF/STS, but not ranked BO, scores.

**Table 4:** Multivariate analyses using Pro.QOL sores as outcome variables: Rank ANCOVA

	Ranked Pro.QOL-BO	Ranked Pro.QOL-CF/STS
R	0.069	0.137
Adjusted R square	0.054	0.123
F (6,373)	**4.628,** ***p*** **< 0.001**	**9.871,** ***p*** **< 0.001**
Ranked AQ	**β = 0.176,** ***p*** **< 0.001**	**β = 0.186,** ***p*** **< 0.001**
Ranked ASRS	β = 0.320, *p* = 0.135	**β = 0.946,** ***p*** **< 0.001**
Ranked Experienced Years	β = 0.005, *p* = 0.861	β = 0.003, *p* = 0.913
Ranked Hospital Bed Number	β = 0.005, *p* = 0.717	β = 0.007, *p* = 0.676
Ranked Interpersonal Work Hours per week	β = 0.005, *p* = 0.856	β = 0.059, *p* = 0.053
Gender	F(1,373) = 0.127, *p* = 0.721	F(1,373) = 1.912, *p* = 0.168

Beta values are standardized coefficients

bold: *p* < 0.05

*Pro.QOL* the professional quality of life scale, *BO* burnout, *CF/STS* compassion fatigue/secondary traumatic stress, *AQ* the autism-spectrum quotient, *ASRS* the adult ADHD self-report scale v-1.1

## Discussion

Our data indicate that there is a relatively high prevalence of psychological distress and burnout/CF among hospital pharmacists. To the best of our knowledge, this is the first report to investigate the association between characteristics that are hypersensitive to interpersonal communication and psychological distress among hospital pharmacists.

As expected, GHQ-12 values were large (54.7 % of our participants obtained a score that was higher than the cutoff point) in our sample population (Table 1). Previous studies using the GHQ-12 have shown various values. In one study, 68.8 % of nurses in general hospitals in Japan [32] obtained a score that was higher than the cutoff point, and first-year medical residents in Japan had a mean score of 4.8 ± 3.2 [33]. However, studies in other countries have reported a lower prevalence of psychological distress as measured by GHQ-12 scores, such as nurses in a Nigerian tertiary health institution (44.1 %) [34] and randomly sampled community pharmacists in New Zealand (40.7 %) [35]. Reports from Japan show a tendency of a high GHQ-12 score. Therefore, cultural differences may exist. We found a relatively high level of burnout/CF, where 49.2 % of the individuals in our sample were facing a high risk of burnout, and 29.2 % were at high risk of CF (Table 1). This result has important implications for the prevalence of psychological distress, and is comparable with previous investigations of other professional groups, such as inpatient oncology staff who have a burnout rate of 44 % and a 37 % rate of CF [7]. The high prevalence of psychological distress, as measured by the GHQ-12, may have been caused not only by interpersonal work, but also by other work-related and daily personal stress. Therefore, we used the GHQ-12 in combination with the Pro.QOL, which specializes in work-related stress. Similar results are obtained from these two measures. Therefore, many pharmacists might be considered to have the risk of a psychological crisis, possibly due to work-related interpersonal stress in addition to conventional occupational stress [5].

GHQ-12 scores were significantly negatively correlated with years of experience (Table 2). This finding is consistent with a study reporting that work-related stress in healthcare workers decreases with years of experience [36]. Therefore, burnout may be prevented through experience and education. However, because this study was a cross-sectional survey, we cannot make statements about causality. Pharmacists without these traits might be more likely to continue to work, while pharmacists who have experienced burnout leave the profession. Because years of experience showed no correlation with burnout, preventive interventions for burnout should target all hospital pharmacists.

As expected, ALT and ADHD symptoms were significantly associated with psychological distress (Table 3). This finding may reflect difficulty not only with work-related stress but also with everyday life stress. Additionally, while the strength of ALT symptoms significantly affected burnout/CF severity, the number of ADHD symptoms only affected CF among hospital pharmacists (Table 4). The reason for this finding may be because ASD is associated with more severe deficits in social cognition [37]. We are confident that our findings regarding burnout and CF reflected psychological stress specific to the workplace because we clearly questioned the participants about their work-related burden as pharmacists (Pro.QOL). Furthermore, we believe that our measure of CF was specific to the interpersonal component of work because the number of hours of interpersonal work per week had a positive, but not significant, effect on CF, while this was not the case for burnout.

Designing burnout preventive interventions specific to individuals with the traits highlighted in this study may be possible. Mindfulness-based interventions are effective burnout protective interventions for primary care physicians [38]. Future research is required to determine whether interventions specific to those with ALT or ADHD symptoms are especially effective.

Individuals with ALTs are often super-moral and have been known to strongly value fairness [39]. Additionally, they generally have an intact capacity to care about others, regardless of their lack of “theory of mind” [39]. Individuals who are vulnerable to burnout in medical practice are also thought to be those who are highly motivated, dedicated, and emotionally involved in their work [40]. Therefore, further research is needed to examine the relationship between occupational burnout and altruistic traits because such characteristics are indispensable for providing high-quality care for patients. More information may lead to the development of programs to help professionals maintain such useful personality traits while avoiding burnout.

This study has several limitations that must be acknowledged. First, in this study, we collected independent and dependent variables by self-report measures without behavioral observations. Therefore, our results might have been strengthened by common method variance. Second, all of the data were collected from one geographic area. Additionally, the sample population was small and the response rate was lower than 50 %. Therefore, selection bias may have occurred. Accumulation of more data from multiple areas is necessary. There may be a limitation to the interpretation of our results, caused by differences between participants and non-participants in the present study. Third, this was a cross-sectional study. In the future, we need to consider the longitudinal turnover rate of pharmacists with ALT or ADHD symptoms. Fourth, we used self-report questionnaires as an index for ALT or ADHD symptoms. Although these scales were validated, the objectivity of answers could have been biased and we did not make definite diagnoses. Fifth, the AQ is considered to reflect static aspects of personality traits or behavior. Therefore, a high level of burnout/CF or depression may influence answers provided in the AQ. Finally, we did not investigate the presence of personal problems for the consideration of privacy. Therefore, our results of the GHQ-12 may have been greatly affected by daily stress unrelated to pharmacists’ work.

## Conclusions

We found a high prevalence of psychological distress and work-related burnout/CF among hospital pharmacists. Personality traits associated with ALT and ADHD symptoms, which are also common among the general population, were associated with an increased risk of psychological distress and work-related burnout/CF. Early risk assessment and appropriate preventive interventions could protect hospital pharmacists with these traits from burnout. Further investigations are required to clarify what aspects of clinical communication skills, such as making eye contact with the patient, considering how to deliver bad news, and addressing patient’s emotions with empathic responses [41], could become a psychological burden for those with ALT or ADHD symptoms relating to communication.

## Abbreviations

ADHD, attention deficit hyperactivity disorder; ALT, autistic-like traits; AQ, autism spectrum quotient; ASRS, adult ADHD self-report scale; BO, burnout; CF/STS, compassion fatigue and secondary traumatic stress; GHQ, general health questionnaire.
